# Assessing the Perceived Exertion in Elite Soccer Players during Official Matches According to Situational Factors

**DOI:** 10.3390/ijerph17020410

**Published:** 2020-01-08

**Authors:** Javier Raya-González, Daniel Castillo, Javier Yanci, Asier Los Arcos

**Affiliations:** 1 Faculty of Health Sciences, Universidad Isabel I, 09003 Burgos, Spain; rayagonzalezjavier@gmail.com; 2 Physical Education and Sport Department, Faculty of Education and Sport, University of Basque Country (UPV/EHU), 01007 Vitoria, Spain; javier.yanci@ehu.es (J.Y.); asier.losarcos@ehu.es (A.L.A.)

**Keywords:** football, match intensity, quantification, playing time

## Abstract

This study aimed to assess the match perceived exertion (PE) declared by starter and non-starter junior elite soccer players, according to the level of the opponents, and by playing at home or away. Nineteen young soccer players who competed in the Spanish U19 League participated in this study. PE was registered during the entire regular season (30 official matches). Players were grouped by match playing time: starters (players who started the game and played at least 45 min) and non-starters (substitute players who participated for less than 45 min). Moreover, the matches were classified according to the opponent level (i.e., high, medium, or low) and the match location (i.e., home or away). Starters who competed against high-level opponents (8.7 ± 0.6) declared higher PE ratings than against medium (8.1 ± 0.7, *p* < 0.01) and low (8.4 ± 0.7, *p* < 0.01) level opponents. In addition, starters competing at home declared lower PE ratings than when playing away (8.2 ± 0.8 vs. 8.5 ± 0.6, *p* < 0.01). However, no significant differences (*p* > 0.05) were observed for non-starters. Coaches should consider not only tactical–strategic needs, but also these contextual factors when managing the match playing time of the starter players.

## 1. Introduction

The training process in soccer is considered to be an effective means for the soccer player to achieve a state of physical performance suitable for the development of the game model proposed by the coach [1]. Therefore, the challenge for physical trainers is to obtain an optimal performance level from players in official matches. Competition has high physical requirements; for example, during an official match, professional soccer players cover approximately 1000 m at high intensity (>18 km/h) and 250 m at a sprint (>21 km/h) [2]. In addition, during a soccer match, the percentage of maximum heart rate (HR_max_) reached is close to the anaerobic threshold, usually 80–90% of HR_max_ [3], but with peaks that can reach up to 98% [4]. Several investigations have analyzed the influence of contextual factors (e.g., match location, opponent level, and match status) on these external and internal load indicators in elite soccer players [5,6,7]. However, in youth soccer players, the acquisition of this type of technology is complicated, which makes it difficult to quantify physical and physiological demands during official matches.

Perceived exertion (PE) has been shown to be a useful alternative to solve this problem, mainly due to it being inexpensive, easy to use, and taking little time to process the data [8,9]. In addition, it has been demonstrated in several studies on soccer players of different categories that PE is a valid and reliable tool [10,11,12]. In relation to playing time, only one study has shown that playing more minutes affects the PE declared by the players [13]. In this sense, more information is needed on how playing time affects the PE declared by starters and non-starters. Moreover, using the PE in the academy of elite soccer clubs would help to assess the PE match load (ML) declared by youth soccer players. Consequently, coaches could design the weekly microcycle, including adequate day-to-day training sessions and rest protocols.

In soccer, the influence of various contextual factors, such as match location, playing style, opponent ranking, time of possession, and/or match outcome, among others, could vary the demands encountered by soccer players during the match [6]. This fact could affect the training context because coaches should adapt the weekly training load of subsequent training weeks [14,15]. In this respect, only one study has analyzed the impact of these variables, specifically the match outcome on the PE reported by soccer players [16]. Fessi and Moalla [16] showed that the match PE was higher after losing in comparison to drawing or winning and higher after drawing than after winning. However, to our knowledge, no study has assessed the influence of situational factors of opponent level and match location on the PE declared by soccer players in official matches. Therefore, it would be interesting for soccer coaches to assess the consequences of these situational factors in elite junior soccer players.

Therefore, the aim of this study was to assess the match PE declared by starter and non-starter junior elite soccer players according to the level of the opponent (i.e., high, medium, or low level) and match location (i.e., home or away).

## 2. Materials and Methods

### 2.1. Participants

Nineteen outfield professional soccer players (age: 18.0 ± 0.6 years, height: 177 ± 5 cm, weight: 70.1 ± 6.8 kg, and body mass index: 22.3 ± 1.5 kg m^−2^) participated in the study. Participants belonged to a soccer academy of the Spanish La Liga Club and they competed in the Spanish First Division Under-19 Championship. The inclusion criterion was that players had to be taking part in any match across the season. The team finished the regular season in 7th position of 16 teams. All of the participants were informed of the objectives of the research, participated voluntarily, and had the possibility to withdraw at any time from the investigation without any penalty. All of the participants, or their parents or tutors, provided written informed consent. The study was conducted according to the Declaration of Helsinki, and the protocol was fully approved by the local research ethics committee before recruitment.

### 2.2. Procedures

Match PE data was collected over a 30-week in-season period during the 2016–2017 season. The competition period started (i.e., first competitive match) on 4 September 2016 and ended (i.e., the last competitive match) on 7 May 2017 (i.e., a full competitive season). In order to classify the teams according to the level of the opponent, the qualifying position obtained by the team after the last day of the official league was considered [17]: (a) high-level, the top 5 classified (*n* = 10 matches), (b) medium-level, teams ranked between 6 and 11 (*n* = 10 matches), and (c) low-level, the last 5 classified (*n* = 10 matches). Players were also classified into two groups: (a) starters, players who started the game and played at least 45 min (*n* = 300 occurrences), and (b) non-starters, substitute players who participated in less than 45 min (*n* = 109 occurrences).

### 2.3. Perceived Exertion (PE)

In order to quantify the soccer match PE [13,18], the Foster’s 0–10 scale was used [19]. Players responded to the question “How hard was the match?” 10 min after every match [20]. Players were allowed to mark a plus sign (interpreted as 0.5 points) alongside the integer value [13,21]. The physical trainer was responsible for asking the question of the players. Each player completed the 0–10 scale randomly without the presence of other players and could not see the values of other participants. All players were familiarized with this method during preseason training and friendly matches (six weeks). The match duration excluded the warm-up and halftime rest [13,20].

### 2.4. Statistical Procedures

Standard statistical methods were used for the calculation of the means and standard deviations (SDs). Match-to-match PE variability was calculated by means of the coefficient of variation ((SD/mean) × 100)). All the variables were normally distributed according to the Shapiro–Wilk test, so we used a parametric analysis. Independent paired *t*-tests were used to determine if any significant differences existed between the PE declared between starters and non-starters. The two-way ANOVA with the Bonferroni post-hoc test was used to assess the impact of the opponent level, the match location, and the interaction of both factors on the PE declared by starter and non-starter players. Practical differences were calculated using Cohen’s *d* effect size (ES, large: >0.8; moderate: between 0.8 and 0.5; small: between 0.5 and 0.2; and trivial <0.2) [22]. The data analysis was carried out using the Statistical Package for Social Sciences (SPSS 21.0, IBM Corp., Armonk, NY, USA). Statistical significance was set at *p* < 0.05.

## 3. Results

**Figure 1:**
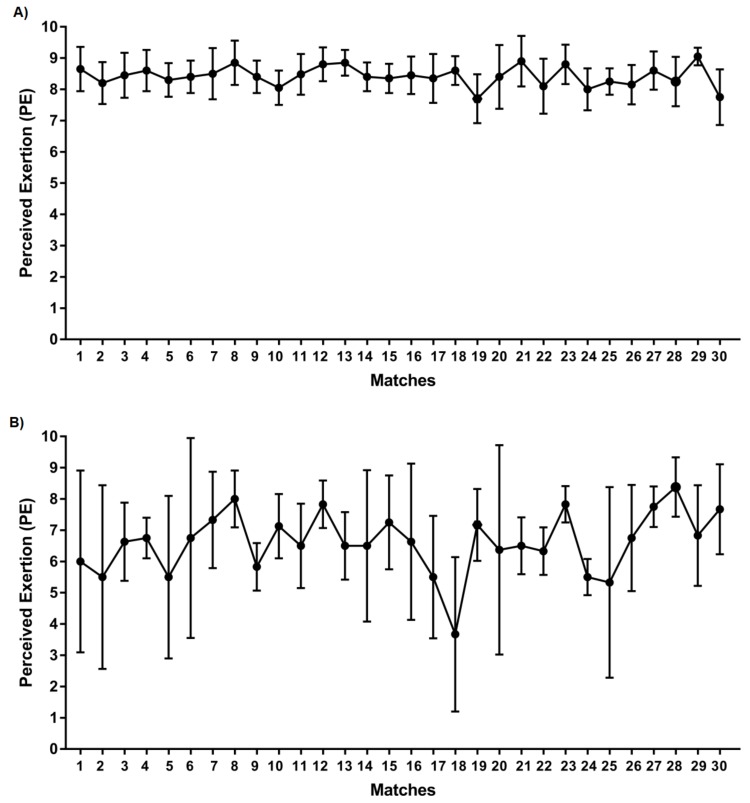
Perceived exertion (PE) declared by starters (**A**) and non-starters (**B**) during official matches along the season.

Starters (82 ± 13 min) declared a higher PE (8.6 ± 0.7) than non-starters (23 ± 13 min; PE = 6.7 ± 2.0) (*p* < 0.01, ES = 0.95, large) during official matches. Figure 1 shows the pattern of PE declared by starter (A) and non-starter (B) players in each match during all competitive season matches (30 matches). Match-to-match PE variability was 7.6 ± 2.4% for starters and 26.0 ± 17.6% for non-starters.

**Figure 2:**
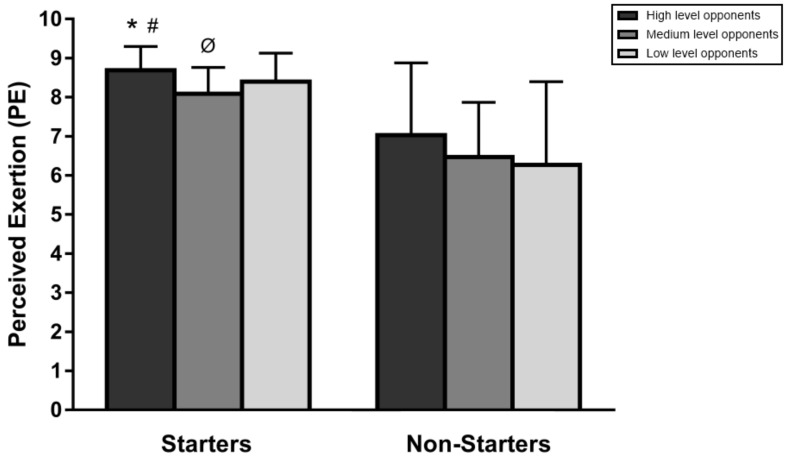
PE for starters and non-starters attending to the level of the opponents. * Significant differences between high- and medium-level opponents (*p* < 0.01), ^#^ Significant differences between high- and low-level opponents (*p* < 0.01), ^Ø^ Significant differences between medium- and low-level opponents (*p* < 0.01).

Starters declared a higher PE (8.7 ± 0.6) after competing against high-level opponents than after competing against medium (8.1 ± 0.7, *p* < 0.01, ES = 0.86, large) or low (8.4 ± 0.7, *p* < 0.01, ES = 0.43, small) level opponents (Figure 2). In addition, the PE declared by players was higher (*p* < 0.01, ES = 0.43, small) after competing against low-level opponents in comparison to medium-level opponents. However, no significant differences (*p* > 0.05) were observed in the PE declared by non-starters when playing against opponents of different levels.

**Figure 3:**
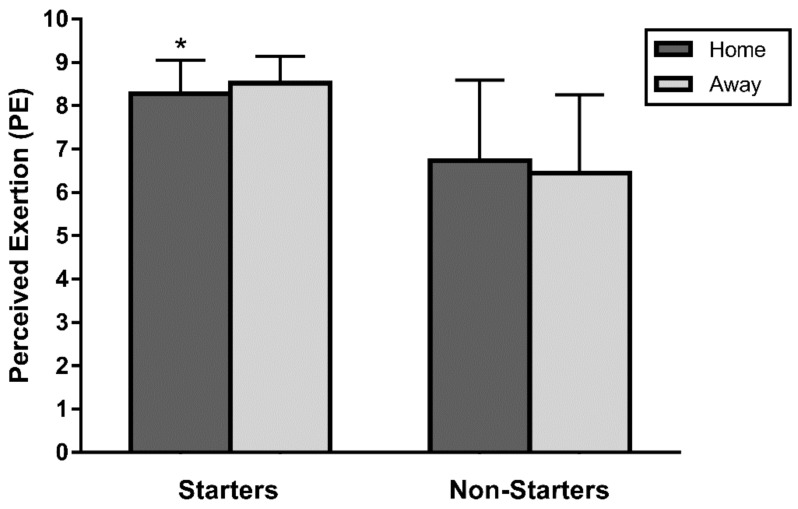
PE for starters and non-starters attending to playing at home or away. * Significant differences between home and away matches (*p* < 0.01).

Starters declared a lower PE when competing at home than when playing away (8.3 ± 0.8 vs. 8.5 ± 0.6, *p* < 0.01, ES = 0.33, small, Figure 3). However, no significant differences (*p* > 0.05) were observed for non-starters regardless of playing at home or away. A two-way ANOVA revealed no significant differences (*p* > 0.05) in PE in the interaction of the factors “level of the opponents” and “playing at home or away” for either starters or non-starters.

## 4. Discussion

The aim of this study was to assess the match PE declared by starter and non-starter junior elite soccer players according to the level of the opponents and playing at home or away. The main findings were: (1) starter and non-starter Spanish junior elite soccer players rated the match as very hard (PE = 8.6 ± 0.7) or hard (PE = 6.7 ± 2.0), respectively; and (2) the competition level of the opponents and playing at home or away affected the match PE declared by starter players; this did not influence the non-starters.

In comparison to English junior elite soccer players that played the entire match (90 min) (PE = 8.4 ± 0.6) [23], starter Spanish junior elite players declared a similar match PE (8.6 ± 0.7). However, these values were higher than the differentiated match PE declared by young professional senior players after playing 90 min (respiratory PE = 6.7 ± 1.3; muscular PE = 6.9 ± 1.6) [13], and by professional soccer players belonging to Qatar´s Stars League that played more than 80 min (≈6.5) [16]. Similarly, non-starter players declared a lower overall PE (6.7 ± 2.0) than young Spanish professional senior soccer players (respiratory PE = 4.6 ± 1.5; muscular PE = 4.1 ± 1.6) [13]. This finding could be explained by the greater high-speed running and sprinting distance imposed on starter players in comparison to non-starter ones in junior soccer players [24]. Therefore, the coaches of elite junior teams should consider that the game is harder for their players than for senior professional players when planning the training strategies of the previous and subsequent weeks. On the other hand, and as expected and found previously in several studies [9,13,25], the starter players declared a significantly higher PE than the non-starters (8.6 ± 0.7 vs. 6.7 ± 2.0, *p* < 0.01; ES = 0.39) due to a greater number of playing minutes. Thus, match playing time can determine the match load and consequently the weekly training load of the players [25].

Despite several studies having assessed match external load variability in soccer players [26,27,28], few studies have assessed the match-to-match PE variability [13]. In line with Los Arcos et al. [13], the between-match PE variability was lower (coefficient of variation (CV) = 7.6 ± 2.4%) for starters than for non-starters (CV = 26.0 ± 17.6%). This suggests that, in addition to playing time, the match PE declared by each player should be considered in order to design post-match training sessions [13]. Moreover, the variability values were lower in starters (CV, 7.6% vs. ≈14%) but similar in non-starters (CV, 26% vs. ≈25%) in comparison to senior professional soccer players [13]. It seems that elite junior matches are harder and more stable than professional soccer matches. Other investigations, focused on external loading, observed a CV of 18.1 and 37.7% in high-intensity running (19.8–25.2 km·h^−1^) and sprinting speeds (>25.2 km·h^−1^), respectively [26].

Several investigations have analyzed the influence of match location, opponent level, and match status on the external load indicators during official matches [5,6,7], but few studies have investigated the effects of these factors on the PE. While the level of the opponents did not affect the match PE of non-starter players, the starter players of the medium competition level team declared a higher match PE (*p* < 0.01) after the matches played against high-level opponents in comparison to medium- and low-level opponents (Figure 1). Moreover, the match PE was higher when playing against lower level opponents than when playing against medium-level opponents. Similarly, the match location did not influence the match PE of non-starter players, but the starters declared a greater PE (*p* < 0.01) when they disputed the matches away compared to when they played at home (PE = 8.5 ± 0.6 vs. 8.3 ± 0.8). Additionally, no interaction effect on the PE declared by players between the opponent ranking of the teams faced and match location was found. From a practical approach, it would be necessary to analyze the influence of these contextual factors separately.

Attending to the aforementioned findings, these contextual factors only have an individual effect when soccer players participate in a large number of minutes in the match, as it is harder to play against high-level teams and away from home. This suggests that the post-match recovery session should be different according to the level of the adversary team and the match location. The contents of this session should be adapted according to the PE match load declared by each player.

## 5. Conclusions

In summary, the study analyzed the effects of the level of the opponents and match location on the match PE declared by young elite soccer players. Soccer situational variables only affected the match PE of the players that almost completed the matches. Specifically, starter players declared higher match PE when they played against teams of different competition levels, as well as when the matches were played away. However, there were no significant differences in PE in the interaction of the two mentioned contextual factors for either starters or non-starters. Coaches should consider not only tactical–strategic needs, but also these contextual factors when managing the match playing time of the starter players. Moreover, due to the influence of the situational factors on the PE declared by starters, the coaches should take into account the level of the opponent and the match location when planning post-match recovery strategies, as well as for the distribution of the weekly training load.
